# Left and right ventricular dyssynchrony and strains from cardiovascular magnetic resonance feature tracking do not predict deterioration of ventricular function in patients with repaired tetralogy of Fallot

**DOI:** 10.1186/s12968-016-0268-8

**Published:** 2016-08-22

**Authors:** Linyuan Jing, Gregory J. Wehner, Jonathan D. Suever, Richard J. Charnigo, Sudad Alhadad, Evan Stearns, Dimitri Mojsejenko, Christopher M. Haggerty, Kelsey Hickey, Anne Marie Valente, Tal Geva, Andrew J. Powell, Brandon K. Fornwalt

**Affiliations:** 1 Saha Cardiovascular Research Center, University of Kentucky, Lexington, KY USA; 2 Institute for Advanced Application, Geisinger Health System, 100 North Academy Avenue, Danville, PA 17822-4400 USA; 3 Department of Biostatistics, University of Kentucky, Lexington, KY USA; 4 Department of Cardiology, Boston Children’s Hospital, Boston, MA USA; 5 Department of Pediatrics, Harvard Medical School, Boston, MA USA

**Keywords:** Tetralogy of Fallot, Cardiac strain, Dyssynchrony, Cardiovascular magnetic resonance, Congenital heart disease

## Abstract

**Background:**

Patients with repaired tetralogy of Fallot (rTOF) suffer from progressive ventricular dysfunction decades after their surgical repair. We hypothesized that measures of ventricular strain and dyssynchrony would predict deterioration of ventricular function in patients with rTOF.

**Methods:**

A database search identified all patients at a single institution with rTOF who underwent cardiovascular magnetic resonance (CMR) at least twice, >6 months apart, without intervening surgical or catheter procedures. Seven primary predictors were derived from the first CMR using a custom feature tracking algorithm: left (LV), right (RV) and inter-ventricular dyssynchrony, LV and RV peak global circumferential strains, and LV and RV peak global longitudinal strains. Three outcomes were defined, whose changes were assessed over time: RV end-diastolic volume, and RV and LV ejection fraction. Multivariate linear mixed models were fit to investigate relationships of outcomes to predictors and ten potential baseline confounders.

**Results:**

One hundred fifty-three patients with rTOF (23 ± 14 years, 50 % male) were included. The mean follow-up duration between the first and last CMR was 2.9 ± 1.3 years. After adjustment for confounders, none of the 7 primary predictors were significantly associated with change over time in the 3 outcome variables. Only 1–17 % of the variability in the change over time in the outcome variables was explained by the baseline predictors and potential confounders.

**Conclusions:**

In patients with repaired tetralogy of Fallot, ventricular dyssynchrony and global strain derived from cine CMR were not significantly related to changes in ventricular size and function over time. The ability to predict deterioration in ventricular function in patients with rTOF using current methods is limited.

**Electronic supplementary material:**

The online version of this article (doi:10.1186/s12968-016-0268-8) contains supplementary material, which is available to authorized users.

## Background

Surgical repair of tetralogy of Fallot (TOF) in early childhood has achieved great success with a low mortality of less than 3 % [1]. However, the mortality rate more than triples 20–30 years after surgery, mostly due to adverse cardiac events [1]. While the use of an outflow tract patch during the repair has been associated with increased late mortality [1], it cannot completely predict which patients are at risk. This increased late mortality has also been linked to pulmonary regurgitation (PR) and volume overload induced by the initial surgery, which leads to right ventricular (RV) dilation, RV dysfunction and sometimes left ventricular (LV) dysfunction [1–3]. Further, there is growing evidence of a link between progressive ventricular dilation/dysfunction and adverse outcomes such as death or sustained ventricular tachycardia in these patients [4–7].

Not all patients with repaired TOF (rTOF) develop progressive dysfunction in the setting of chronic PR. Unfortunately, no clinical measures have been reported to predict deterioration in function and dilation. For example, a recent study by Wald et al. [8] investigated clinical, electrocardiographic (ECG), exercise, and cardiovascular magnetic resonance (CMR) parameters in a large cohort of patients with rTOF. The authors failed to identify any metrics that predicted significant deterioration in ventricular function in patients with rTOF. However, the ability of myocardial strain or dyssynchrony parameters to predict deterioration in ventricular function in patients with rTOF was not assessed.

Measures of cardiac mechanics, such as ventricular dyssynchrony and strain, are strong predictors of adverse outcomes in patients with cardiovascular disease [9, 10]. Patients with rTOF suffer from intra- (LV and RV) and inter- ventricular dyssynchrony [11–16], and impairment in myocardial strain [15, 17–19]. However, the role of ventricular dyssynchrony and strain in predicting deterioration of ventricular dilation and dysfunction in patients with rTOF remains unknown. We hypothesized that the presence of dyssynchrony and decreased strain, derived from standard cine CMR using a feature-tracking based method, would predict deterioration in ventricular size and function in patients with rTOF.

## Methods

### Patient enrollment

Patients fulfilling the following criteria were retrospectively identified from a database search at Boston Children’s Hospital: 1) diagnosis of rTOF; 2) at least 2 CMR examinations with assessment of ventricular size and function greater than 6 months apart, acquired between May 2005 and March 2012; 3) no intervening surgery or catheter procedures between the CMR examinations; 4) a 12-lead ECG at the time of first CMR. If a patient had at least 2 CMR scans before an intervening procedure, their data were included in the study up to the point of the procedure. Patients with incomplete or poor quality CMR were excluded.

### Standard imaging protocol

CMR was performed on a 1.5 T Philips Achieva scanner with a 32-element phased array cardiac coil (Philips Medical Systems, Best, the Netherlands). ECG-gated steady-state free precession (SSFP) short-axis images spanning the ventricles were acquired during 10–15 s breath holds with 20–30 image frames per cardiac cycle. Acquisition parameters were: matrix 256 x 256, field of view 240–460 mm^2^, flip angle 60°, TR 2.7–3.6 ms, TE 1.4–1.8 ms, slice thickness 6–8 mm, slice gap 0–2 mm. Horizontal and vertical long-axis images were used for identification of valve planes. Cine phase-contrast images perpendicular to the main PA were used for quantification of the PR fraction.

### Data analysis

LV and RV end-diastolic (EDV) and end-systolic volumes (ESV), and ejection fractions (EFs) were quantified from all CMR scans using Qmass (Medis medical imaging systems, Leiden, the Netherlands) as previously described [20]. Ventricular volumes were indexed to body surface area and z-scores were computed based on normative data [20]. QRS duration and heart rate were measured from the 12-lead ECG at the time of the first CMR study (baseline).

**Fig. 1:**
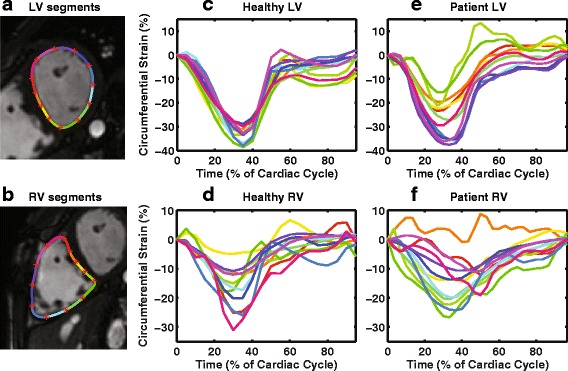
Representative short-axis images with LV and RV segments (**a, b**) and segmental circumferential strain curves in a healthy subject (**c, d**) and a patient with rTOF (**e, f**)

CMR images from the baseline study were used to quantify cardiac dyssynchrony and strain using custom feature tracking software written in MATLAB (The Mathworks, Natick, MA, USA). A detailed description of the method was reported in a previous study [16]. Briefly, short-axis images located between the LV/RV apex and the mitral/tricuspid valve plane (identified from the end-systolic long-axis four-chamber image) were selected for post-processing. Approximately 4–8 slices were analyzed for each patient. Endocardial borders for both ventricles were semi-automatically identified. Circumferential strain curves were generated for 12 segments around each ventricle for each short-axis slice using a displacement-based feature-tracking algorithm (Fig. 1). Longitudinal strain curves were generated using a four-chamber long-axis slice.

To quantify dyssynchrony, cross-correlation delays for each of the segmental strain curves (ranging from 48 to 96 total segments for each ventricle based on the number of short-axis slices available) were calculated relative to a patient-specific reference curve derived from the LV of each patient [16]. Intra-ventricular (LV and RV) dyssynchrony was defined as the standard deviation (SD) of the delays within each ventricle, and inter-ventricular dyssynchrony was defined as the difference between the median delays of the LV and the RV [16].

Peak circumferential strain of each ventricle was calculated as the most negative peak of the global strain curve obtained by averaging all segmental strain curves across all selected short-axis slices. Similarly, peak longitudinal strain of each ventricle was calculated from a four-chamber slice. Absolute values of the peak strains were reported.

### Predictors, outcomes and confounders

Three primary outcomes were defined to measure deterioration in ventricular size and function over time: RVEDV indexed to body surface area, RVEF and LVEF. Changes in the outcomes are represented as ΔRVEDVi, ΔRVEF and ΔLVEF, respectively. Seven potential primary predictors were defined at baseline: LV, RV and inter-ventricular dyssynchrony, LV and RV peak circumferential strains, and LV and RV peak longitudinal strains. Ten potential confounders measured at baseline were also included in the analysis: RVEDVi, RVEF, LVEF, indexed RVESV (RVESVi), PR fraction, QRS duration, heart rate, age at first CMR, age at initial repair, and type of initial repair (categories listed in Table 1). Note that the three outcomes measured at baseline (RVEDVi, RVEF and LVEF) were also included as potential confounders.

**Table 1:** Baseline demographic, electrocardiographic and CMR parameters of the study subjects (*n* = 153)

Variables	Mean ± SD (z-score) or N (%)
Male	76 (50 %)
Body surface area (m^2^)	1.6 ± 0.4
Age at CMR (years)	23 ± 14
Age at surgical repair (years)	3.5 ± 6.7
Type of initial repair	
Transannular patch	97 (63 %)
RV-PA conduit	11 (7 %)
RVOT patch, non-transannular	30 (20 %)
Infundibular resection & pulmonary valvotomy	2 (1 %)
Pulmonary valvotomy alone	9 (6 %)
Other	4 (3 %)
Electrocardiogram	
QRS duration (ms)	136 ± 27
Heart rate (beats/min)	80 ± 17
CMR parameters	
Pulmonary regurgitation fraction (%)	36 ± 15
Indexed LV end-diastolic volume (mL/m^2^)	85 ± 15 (-0.7 ± 1.6)
Indexed LV end-systolic volume (mL/m^2^)	35 ± 9 (0.0 ± 1.6)
LV ejection fraction (%)	59 ± 6 (-1.0 ± 1.3)
Indexed RV end-diastolic volume (mL/m^2^)	143 ± 36 (2.6 ± 2.8)
Indexed RV end-systolic volume (mL/m^2^)	67 ± 23 (3.2 ± 3.6)
Indexed RV stroke volume (mL/m^2^)	76 ± 20 (1.7 ± 2.3)
RV ejection fraction (%)	53 ± 8 (-1.0 ± 1.8)
LV dyssynchrony (ms)	19 ± 11 (1.6 ± 2.1)
RV dyssynchrony (ms)	57 ± 29 (0.6 ± 1.7)
Inter-ventricular dyssynchrony (ms)^a^	-40 ± 20 (-1.8 ± 0.7)
LV circumferential strain (%)	27 ± 3 (-0.6 ± 1.0)
RV circumferential strain (%)	18 ± 3 (1.2 ± 1.1)
LV longitudinal strain (%)	19 ± 3 (-1.0 ± 1.2)
RV longitudinal strain (%)	23 ± 3 (-0.7 ± 1.6)

^a^Negative values of inter-ventricular dyssynchrony represent delayed contraction in the right ventricle

### Statistics

Statistical analyses were performed in SAS Version 9.3 (SAS Institute Inc, Cary, NC, USA) and MATLAB. The three outcomes (ΔRVEDVi, ΔRVEF and ΔLVEF measured across multiple CMR scans) were treated as continuous variables and analyzed with linear mixed models to assess how their baseline levels and changes over time were associated with various potential predictors and confounders. A primary multivariate analysis with the same outcomes was also performed, in which multiple potential predictors and confounders and their interactions with time were included simultaneously after selection by a backward elimination process. Normalized mixed model coefficient estimates (β, referred to as “coefficients” hereafter) are reported. The normalized coefficients are roughly comparable to the linear correlation coefficient r. An auxiliary multivariate analysis was performed to estimate the proportions of variability in the outcomes’ changes over time which were accounted for by covariates in the primary multivariate analysis; the auxiliary multivariate analysis replaced the outcomes by difference quotient approximations to their time derivatives and included only those covariates interacting with time in the primary multivariate analysis. Statistical significance was defined by *p*-values < 0.05. Data are summarized by mean ± SD or number (percent), with two-group comparisons based on T tests or Fisher’s exact test. Post-hoc linear regressions were also fit to assess change over time in relation to time elapsed for selected outcomes, overall and within strata defined by quartiles of baseline predictors.

A subgroup analysis was conducted between patients with pronounced deterioration in ventricular function and/or worsening ventricular dilation and patients who had no deterioration. Pronounced deterioration was defined as the fulfillment of any of the following criteria: 1) increase in RVEDVi ≥30 mL/m^2^, 2) decrease in RVEF ≥10 absolute percentage points, or 3) decrease in LVEF ≥10 absolute percentage points. Conversely, patients fulfilling all three of the following criteria were classified as no deterioration: 1) increase in RVEDVi ≤5 mL/m^2^, 2) decrease in RVEF ≤3 absolute percentage points, and 3) decrease in LVEF ≤3 absolute percentage points. Threshold values were selected based on a previous study [8]. Unpaired t-tests were used for statistical analysis.

Because the assessment of global function, including peak global strains, may mask potentially important regional dysfunction, the entire analysis was repeated by replacing the above peak global strains with peak regional strains. For each ventricle, peak circumferential strain was assessed in nine regions while longitudinal strain was assessed in two regions from the four-chamber image.

## Results

### Characteristics of study subjects

The database search identified 164 patients based on the inclusion criteria, among which 4 had incomplete imaging data, and 7 were excluded due to poor image quality. A total of 153 patients with rTOF (23 ± 14 years, 50 % male) were included for analysis. Demographic, surgical and ECG data are summarized in Table 1. On average, each patient had 2.4 CMR scans (range 2–6), with an average follow-up duration of 2.9 years (range 6 months–5.9 years). Patients underwent initial surgical repair at 3 ± 7 years old with a transannular patch approach being the most common (*n* = 97). All patients were in normal sinus rhythm, and the mean QRS duration (136 ± 27 ms) was prolonged. Ninety-two percent (*n* = 140) of the patients had right bundle branch block on ECG.

### Baseline CMR parameters and outcomes

CMR parameters evaluated at the baseline scan are listed in Table 1. On average, patients had slightly reduced LVEF (z-score: -1.0 ± 1.3) and RVEF (z-score: -1.0 ± 1.8). LVEDV and ESV were normal but both RVEDV and RVESV were enlarged (z-scores 2.6 ± 2.8 and 3.2 ± 3.6, respectively). The mean PR fraction was 36 ± 15 %.

Results of the seven primary predictors are included in Table 1. Compared to a group of healthy normal volunteers from a previous study with identical methodology [16], patients with rTOF had LV dyssynchrony (z-score: 1.6 ± 2.1), RV dyssynchrony (z-score: 0.6 ± 1.7) and inter-ventricular dyssynchrony (z-score: -1.8 ± 0.7) with delayed RV contraction. Patients also had decreased LV circumferential strain (z-score: -0.6 ± 1.0) and increased RV circumferential strain (z-score: 1.2 ± 1.1). Both LV and RV longitudinal strain were reduced in the patients (z-score: -1.0 ± 1.2 and -0.7 ± 1.6, respectively). Strain results from the group of healthy normal volunteers from the previous study are included in (Additional file 1: Table S1). Inter-test reproducibility of peak global and regional strains from the previous study are shown in (Additional file 2: Table S2) [16].

**Fig. 2:**
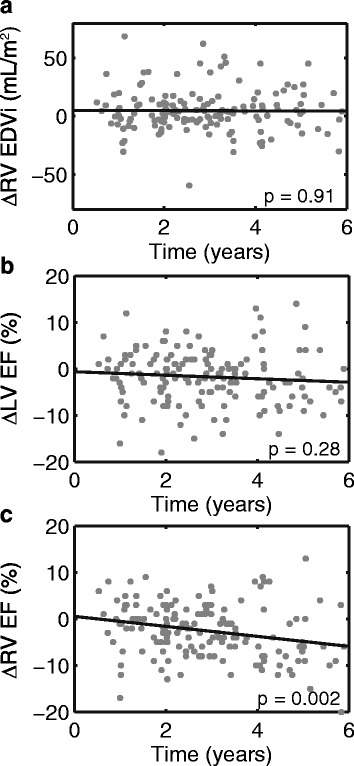
Changes in indexed right ventricular (RV) end-diastolic volume (EDVi) (**a**), left ventricular (LV) ejection fraction (EF) (**b**) and RVEF (**c**) over the study period. ∆RVEDVi, ∆LVEF, ∆RVEF are computed as the difference between the first and last CMR study in each patient. Note that changes in EF are in units of absolute, not relative, percentage points

Change in ventricular size and ejection fraction computed between the first and last CMR scans are summarized in Table 2. During the study period, patients on average had a small increase in RVEDVi (5 ± 18 mL/m^2^), and small decreases in LVEF (2 ± 6 %) and RVEF (3 ± 6 %). The mean rate of change per year was 2 ± 10 mL/m^2^ for RVEDVi, -1 ± 3 % for LVEF and -1 ± 3 % for RVEF (Fig. 2). ΔRVEF was the only outcome variable that changed significantly over time (*p* = 0.002).

**Table 2:** Changes in ventricular size and function over the study period (*n* = 153)

	Mean difference ± SD over entire study^a^	Mean change per year
∆RVEDVi (mL/m^2^)	5 ± 18	2 ± 10
∆RVEF (%)	-3 ± 6	-1 ± 3
∆LVEF (%)	-2 ± 6	-1 ± 3

^a^Difference was computed between the first and last CMR scans

### Correlation of primary predictors with outcomes before adjusting for confounders

**Fig. 3:**
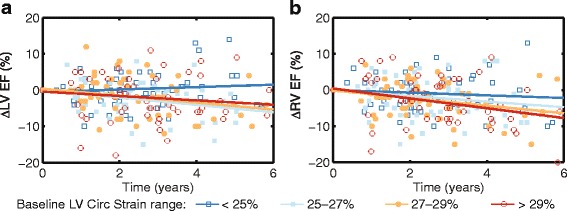
Changes in LV (**a**) and RV (**b**) ejection fraction (EF) over time, with patients divided into 4 groups based on the four quartiles of baseline LV peak circumferential strain of the patients. ∆LVEF and ∆RVEF were calculated with respect to the baseline value for each subject. A straight line was fitted to each group using linear regression to facilitate visualizing group differences in changes in EF over time

Table 3 includes the results of the linear mixed models between the seven primary predictors and the three outcomes. None of the three dyssynchrony measures were significantly associated with changes in RVEDVi, RVEF, and LVEF over time. LV peak circumferential strain was weakly associated with ∆LVEF (β = -0.09, *p* < 0.001) and ∆RVEF (β = -0.06, *p* < 0.001); however, the direction of correlation was negative (Table 3, Fig. 3). For example, Fig. 3a shows that for patients with the most impaired LV circumferential strain at baseline, LVEF was stable or even slightly improved over time. Conversely, LVEF tended to decrease over time for patients with baseline LV circumferential strains in the higher quartiles. Similarly, both LV and RV longitudinal strains were weakly correlated with both ∆LVEF and ∆RVEF, all in negative directions (Table 3).

**Table 3:** Correlation between primary predictors and outcomes before adjusting for confounders (*n* = 153)

	∆RVEDVi		∆RVEF		∆LVEF	
	β	*p*	β	*p*	β	*p*
LV dyssynchrony	0.01	0.41	-0.02	0.39	0.02	0.43
RV dyssynchrony	-0.004	0.77	-0.01	0.62	0.004	0.86
Inter-ventricular dyssynchrony	-0.001	0.91	0.001	0.98	0.009	0.71
LV circumferential strain	0.01	0.28	**-0.06**	**<0.001**	**-0.09**	**<0.001**
RV circumferential strain	0.02	0.18	-0.03	0.09	-0.04	0.12
LV longitudinal strain	0.01	0.30	**-0.04**	**0.01**	**-0.05**	**0.03**
RV longitudinal strain	-0.002	0.87	**-0.05**	**0.02**	**-0.05**	**0.04**

Correlations with statistical significance (*p*<0.05) are shown in bold

### Correlation of other predictors with outcomes before adjusting for confounders

**Fig. 4:**
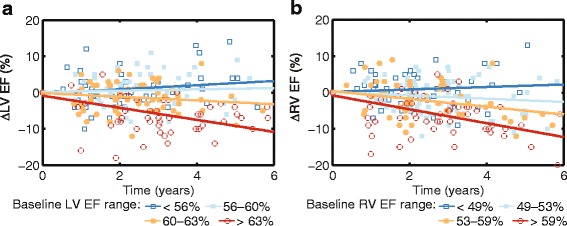
Association of baseline left ventricular (LV) ejection fraction (EF) with ∆LVEF (**a**), and baseline RVEF with ∆RVEF (**b**). The patients are divided into four groups based on the four quartiles of baseline EFs

To investigate the correlation between the baseline confounders and the three outcomes, we also fit linear mixed models using each of the confounders. Of the baseline confounders, only LVEF, RVEF and type of initial repair were related to changes in ejection fraction over time (Table 4). Baseline LVEF and RVEF were both negatively associated with ∆LVEF and ∆RVEF (Fig. 4) indicating that patients with better EFs at baseline have more deterioration in EF over time.

**Table 4:** Correlation between other predictors and outcomes before adjusting for confounders (*n* = 153)

	∆RVEDVi		∆RVEF		∆LVEF	
	β	*p*	β	*p*	β	*p*
LVEF	0.01	0.19	**-0.10**	**<0.001**	**-0.15**	**<0.001**
RVEF	0.004	0.68	**-0.12**	**<0.001**	**-0.06**	**0.009**
Type of Repair^a^	NA	0.52	NA	**0.005**	NA	0.44
RVEDVi	0.01	0.37	-0.02	0.28	-0.02	0.56
RVESVi	0.008	0.55	0.03	0.11	0.009	0.73
PR Fraction	0.01	0.25	-0.02	0.38	0.02	0.48
Heart Rate	0.002	0.83	0.03	0.15	0.03	0.13
Gender	0.01	0.56	-0.005	0.89	-0.05	0.30
QRS Duration	-0.004	0.74	-0.008	0.66	-0.004	0.88
Age at Repair	0.005	0.59	-0.004	0.80	0.002	0.92

^a^The *p*-value represents the statistical significance of a set of regression coefficients associated with various repair types

Correlations with statistical significance (*p*<0.05) are shown in bold

### Multivariate analysis

A summary of the results from multivariate analysis is shown in Table 5. All seven primary predictors were removed from the multivariate model during backward elimination; therefore, none were significantly associated with ∆RVEDVi, ∆RVEF or ∆LVEF after adjusting for confounders. A few baseline confounders remained in the multivariate models which had significant contributions to deteriorations in the outcome variables. Baseline LVEF was significantly associated with ∆LVEF (β = -0.16, *p* < 0.001) and weakly correlated with ∆RVEF (β = -0.03, *p* = 0.05). Similarly, baseline RVEF was weakly correlated with ∆RVEF (β = -0.13, *p* < 0.001). A similar negative correlation was also seen between baseline QRS duration and ∆RVEF (β = -0.07, *p* < 0.001). However, as quantified by the auxiliary multivariate analysis, covariates in the primary multivariate analysis explained only 1 %, 17 % and 9 % of the variability in ∆RVEDVi, ∆RVEF and ∆LVEF, respectively.

**Table 5:** Summary of results from the multivariate analysis (*n* = 153)

	∆RVEDVi		∆RVEF		∆LVEF	
	β	*p*	β	*p*	β	*p*
RVEF	0.001	0.95	**-0.13**	**<0.001**	-0.02	0.33
LVEF	0.007	0.58	**-0.03**	**0.05**	**-0.16**	**<0.001**
QRS duration	0.005	0.71	**-0.07**	**<0.001**	-0.008	0.72

Correlations with statistical significance (*p*<0.05) are shown in bold

### Subgroup analysis

**Fig. 5:**
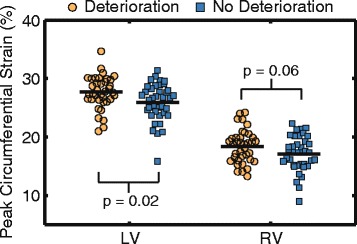
LV and RV peak circumferential strain in patients with pronounced deterioration (*n* = 37) and patients with no deterioration (*n* = 38)

Out of 153 patients in the study, 37 (24 %) patients had pronounced deterioration and 38 (25 %) patients had no deterioration. Seventy-eight patients did not fulfill criteria for either group (i.e. their deterioration was not pronounced). Between the 2 groups, there were no differences in measures of dyssynchrony or longitudinal strains (Table 6). Patients with pronounced deterioration had higher baseline LV circumferential strain compared to those without deterioration (28 ± 3 % vs 26 ± 3 %, *p* = 0.02, Fig. 5). Similarly, patients with pronounced deterioration tended to have higher RV circumferential strain (18 ± 3 % vs 17 ± 3 %, *p* = 0.06, Fig. 5). These results are consistent with the negative correlation observed between strains and ∆EFs in the linear mixed models.

**Table 6:** Comparison of baseline parameters (mean ± SD) between patients with and without deterioration

Baseline variables	Deterioration	No deterioration	*p*-value
	(*n* = 37)	(*n* = 38)	
Male	20 (54 %)	20 (53 %)	0.90
Age at repair (years)	4.4 ± 9.1	4.3 ± 8.5	0.54
Type of repair			**0.03**
Transannular patch	24 (65 %)	19 (50 %)	
RV-PA conduit	2 (5 %)	5 (13 %)	
RVOT patch non-transannular	5 (14 %)	11 (29 %)	
Pulmonary valvotomy alone	5 (14 %)	2 (5 %)	
Other	1 (3 %)	1 (3 %)	
LVEF (%)	62 ± 6	56 ± 6	**<0.001**
RVEF (%)	56 ± 8	52 ± 7	**0.03**
RVEDVi (mL/m^2^)	153 ± 37	136 ± 34	**0.04**
RVESVi (mL/m^2^)	69 ± 24	64 ± 22	0.29
QRS duration (ms)	138 ± 27	130 ± 25	0.19
Heart rate (beats/min)	78 ± 19	83 ± 21	0.27
PR fraction (%)	40 ± 13	35 ± 15	0.10
LV dyssynchrony (ms)	20 ± 13	20 ± 11	0.99
RV dyssynchrony (ms)	56 ± 29	61 ± 29	0.39
Inter-ventricular dyssynchrony (ms)	-38 ± 19	-39 ± 25	0.74
LV circumferential strain (%)	28 ± 3	26 ± 3	**0.02**
RV circumferential strain (%)	18 ± 3	17 ± 3	0.06
LV longitudinal strain (%)	20 ± 4	19 ± 3	0.29
RV longitudinal strain (%)	23 ± 3	23 ± 3	0.77

Correlations with statistical significance (*p*<0.05) are shown in bold

In addition, patients with pronounced deterioration also had higher baseline LVEF (62 ± 6 % vs 56 ± 6 %, *p* < 0.001), RVEF (56 ± 8 % vs 52 ± 7 %, *p* = 0.03) and RVEDVi (153 ± 37 mL/m^2^ vs 136 ± 34 mL/m^2^, *p* = 0.04) compared to the group without deterioration. QRS duration, heart rate, age at initial repair, PR fraction and RVESVi were no different between the two groups (Table 6). The type of repair was different between the two groups (*p* = 0.03): the deterioration group had more patients with a transannular patch repair and the no deterioration group had more patients with a non-transannular patch repair.

### Regional strain analyses

Baseline peak regional strains are summarized in (Additional file 3: Table S3). After adjusting for confounders and applying backward elimination, one regional strain remained in the model (peak circumferential strain in the basal outflow region of the RV). There was a weak positive correlation between this regional strain and ∆RVEF (β = 0.04, p = 0.01) (Additional file 3: Table S5). Compared to the original multivariate model, this new model was able to explain 1 % more of the variability in each of the outcome variables (for a total of 2 %, 18 % and 10 % of the variability in ∆RVEDVi, ∆RVEF and ∆LVEF, respectively).

## Discussion

We investigated for the first time the relation of feature-tracking based cardiac dyssynchrony and strain derived from standard CMR to the longitudinal changes in ventricular volume and ejection fraction in a large cohort of patients with rTOF. Major findings include: 1) measures of ventricular dyssynchrony are not correlated with changes in ventricular volume and ejection fraction over time; 2) baseline LV and RV peak global circumferential/longitudinal strains correlate weakly and negatively with ∆LVEF and ∆RVEF before adjusting for baseline confounders, but they are not significantly associated with ∆LVEF or ∆RVEF after adjustment for potential confounders; 3) multivariate analysis shows baseline LV and RV EF are correlated to ∆LVEF and ∆RVEF after adjusting for confounders, yet the direction of correlation is negative.

### Progressive dilation and dysfunction portends poor outcomes in patients with rTOF

Progressive RV dilation as well as LV and RV dysfunction are commonly seen in patients with rTOF, and have been identified as independent predictors of adverse clinical status in this patient population [4–7]. Determining predictors of progressive dilation and dysfunction could help identify patients at increased risk of deterioration and in need of more frequent follow-up and/or interventional procedures. A few studies [8, 21, 22] have explored potential predictors (history, ECG, exercise and CMR parameters) of progressive ventricular dilation and dysfunction; however, none were able to identify robust predictors and none included cardiac dyssynchrony and strain as potential predictors.

### Cardiac mechanics as predictors of progressive ventricular dilation and dysfunction

Based on findings that cardiac strain and dyssynchrony are strong predictors of adverse outcomes in other diseases [9, 10], we hypothesized that these metrics would similarly relate to deleterious processes in rTOF. In fact, several studies on small cross-sectional datasets [11, 14, 17, 23–25] have demonstrated potential contributions of cardiac strain and dyssynchrony to ventricular dilation and dysfunction. Most of these studies were also limited by their exclusive focus on LV mechanics despite the fact that RV dysfunction is dominant in TOF. The current study sought to overcome these limitations and rigorously evaluate our hypothesis by investigating the relation of biventricular dyssynchrony and strain to changes in biventricular size and function in a large cohort of patients with rTOF with serial follow-up.

Consistent with previous work, we observed intra- and inter-ventricular dyssynchrony [11–15, 26] as well as altered myocardial strain [17–19, 23] in the current cohort. However, the degree of dyssynchrony was not associated with longitudinal changes in ejection fraction. Moreover, global LV and RV longitudinal strains, as well as LV circumferential strain, were only weakly associated with ∆LVEF and ∆RVEF and only before adjusting for baseline confounders. Furthermore, the strain and EF correlations were negative, indicating that patients with higher strain at baseline have bigger decreases in EFs over time, contrary to our hypothesized relationship. Consistently, the subgroup analysis showed that patients with pronounced deterioration had higher circumferential strain at baseline compared to those without deterioration. The reason for this negative correlation between strain and change in function is unknown. A potential explanation could be the “regression to the mean” phenomenon, since people with higher EF at baseline have more room to fall in a subsequent study. Similar findings have also been shown in a recent study with a larger population in which patients with rTOF with pronounced disease progression had higher LV and RV EF at baseline compared to patients with no deterioration [8].

Finally, after adjusting for baseline confounders, none of the seven primary predictors were significantly associated with ∆RVEDVi, ∆RVEF or ∆LVEF. The ultimate exclusion of global peak strains (circumferential and longitudinal) from the multivariate model was likely due to their tight correlation with LV and RV EF at baseline (data not shown).

### Evaluating other predictors of progressive ventricular dilation and dysfunction

Multivariate analysis showed that baseline LV and RV EFs were significantly correlated with ∆EFs. These correlations were also negative, consistent with the finding from the subgroup analysis. Similarly, Wald et al. [8] reported higher LV and RV EF at baseline in patients with significant deterioration compared to patients without deterioration. This finding further supports previous observations that most patients with rTOF could potentially remain stable without interventions, but the disease progresses rapidly in some patients [21, 22]. Due to the inability to identify any strong predictors for disease deterioration, all patients with rTOF will require frequent follow-up CMR evaluations to monitor clinical status. The optimal follow-up frequency has been shown to be 3 years [8].

In the current study, we did not identify any variables that were independently associated with changes in RVEDVi over time. This could be due to the generally small changes in RVEDVi in this cohort during the study period. In the subgroup analysis, patients with pronounced deterioration had higher RVEDVi at baseline compared to the non-deterioration group. Similarly, Luijnenburg et al. [22] has reported that lower effective RV stroke volume at baseline was related to steeper increase in RVEDV over a period of 5 years. This suggests that patients with a more severely dilated RV may have an increased risk for progressive RV dilation.

The type of the initial repair has been suggested as a predictor for clinical status [3]. Patients who had a transannular patch have been shown to have a higher chance to develop ventricular arrhythmia and sudden cardiac death compared to those with other types of repair such as a non-transannular patch. Consistent with this finding, in the current study, we also observed an association between the transannular patch repair and deterioration. Further, the type of repair was weakly associated with ∆RVEF before adjusting for other confounders. However, the sample size in each repair type was heavily unbalanced, making the statistical results less definitive.

It is worth noting that with all potential predictors and confounders considered, only a few confounders remained in the final multivariate model, and the parameters remaining in the model could explain no more than 17 % of the variations in changes over time in the three outcomes. Even when global strains were replaced with regional strains in the analyses, only one regional strain remained significant and it was weakly correlated to a change in an outcome variable. This finding is likely spurious, as the predictive ability of the model increased by only 1 % upon including this regional strain. This suggests that our ability to predict deterioration of ventricular function in patients with rTOF is limited. There are likely unknown factors that play critical roles in mediating deterioration of the disease, and this demands further investigation.

### Limitations

Patients with interventions were excluded from the current study, which may have limited our study subjects to asymptomatic patients or patients with mild deterioration in ventricular dysfunction. However, those who had at least two CMR scans before any intervention were still included. Also, patients who had contraindications for CMR (e.g., pacemakers) were not able to be included in the study.

Peak strains in the current study were quantified using feature tracking from SSFP images. While feature tracking has shown moderate reproducibility for quantifying RV strains [27] and allows us to utilize the large dataset of existing SSFP images, it may be missing new and valuable information about the disease. More advanced image acquisition techniques, such as myocardial tagging and displacement encoding with stimulated echoes (DENSE), are believed to be the gold standard for measuring mechanics and superior to feature tracking [28]. However, DENSE is a relatively new technique that has not been implemented in the clinical setting. Future studies need to investigate whether dyssynchrony and strain quantified by more advanced techniques are associated with deterioration of cardiac function in patients with rTOF.

## Conclusions

Feature-tracking based measures of cardiac mechanics (dyssynchrony and global strain) do not independently predict changes in ventricular size and ejection fraction over time in patients with repaired tetralogy of Fallot. The ability to predict deterioration in cardiac function using currently available methods is very poor; less than 17 % of the variations in the outcomes could be explained by the multivariate model. These findings suggest that patients with repaired tetralogy of Fallot require similar clinical follow-up, including CMR evaluations to assess ventricular size and ejection fraction, regardless of their clinical status.

## Abbreviations

(r)TOF, (repaired) tetralogy of Fallot; CMR, cardiovascular magnetic resonance; DENSE, displacement encoding with stimulated echoes; ECG, electrocardiogram; EDV(i), (indexed) end-diastolic volume; EF, ejection fraction; ESV(i), (indexed) end-systolic volume; LV, left ventricle/ventricular; PR, pulmonary regurgitation; RV, right ventricle/ventricular; SD, standard deviation; SSFP, steady-state free precession

## Additional files

Additional file 1: Table S1. Peak strains of healthy cohort in a previous study. (DOCX 20 kb) Additional file 2: Table S2. Inter-test reproducibility of peak strains from patient cohort in a previous study. (DOCX 21 kb) Additional file 3: Tables S3-S6. Multivariate analysis with regional strains. (DOCX 28 kb)
