# Supplementary material for: Left and right ventricular dyssynchrony and strains from cardiovascular magnetic resonance feature tracking do not predict deterioration of ventricular function in patients with repaired tetralogy of Fallot
Source: J Cardiovasc Magn Reson. 2016 Aug 22;18:49. doi: 10.1186/s12968-016-0268-8 (PMC4993000; doi:10.1186/s12968-016-0268-8)
Supplement: Additional file 1: Table S1. — Peak strains of healthy cohort in a previous study. (DOCX 20 kb) [file 12968_2016_268_MOESM1_ESM.docx]

**Peak Strains of Healthy Cohort in a Previous Study**

In a previous study with identical image acquisition and image processing methodology, 17 healthy controls were enrolled [1]. Peak global strains from this cohort are reported in Table S1. The mean and standard deviations from this healthy cohort were used to calculate z-scores for the patients in the main manuscript. By deriving strain from the endocardial region of the ventricles, the values for these strains may appear somewhat high, particularly for LV circumferential strain. This is due to the natural gradient in circumferential strain across the wall of the ventricle such that circumferential strain is largest at the endocardium and smallest at the epicardium. A previous study that used feature tracking at the endocardium found similar values for LV circumferential strain in their cohorts of healthy and hypertrophic patients (25.9 ± 3.3% and 33.2 ± 5.0%, respectively) [2].

| **Table S1. Peak Global Strains of Healthy Cohort (n = 17)** |  |
| --- | --- |
| Variables | Mean ± SD |
| LV circumferential strain (%) | 29 ± 3 |
| RV circumferential strain (%) | 14 ± 3 |
| LV longitudinal strain (%) | 22 ± 3 |
| RV longitudinal strain (%) | 24 ± 2 |

**References:**

1. Jing L, Haggerty CM, Suever JD, Alhadad S, Prakash A, Cecchin F, Skrinjar O, Geva T, Powell AJ, Fornwalt BK: **Patients with repaired tetralogy of Fallot suffer from intra- and inter-ventricular cardiac dyssynchrony: a cardiac magnetic resonance study.** *Eur Heart J Cardiovasc Imaging* 2014, **15**:1333–43.

2. Wu L, Germans T, Guclu A, Heymans MW, Allaart CP, van Rossum AC: **Feature tracking compared with tissue tagging measurements of segmental strain by cardiovascular magnetic resonance**. *J Cardiovasc Magn Reson* 2014, **16**:10.
