# Supplementary material for: Left and right ventricular dyssynchrony and strains from cardiovascular magnetic resonance feature tracking do not predict deterioration of ventricular function in patients with repaired tetralogy of Fallot
Source: J Cardiovasc Magn Reson. 2016 Aug 22;18:49. doi: 10.1186/s12968-016-0268-8 (PMC4993000; doi:10.1186/s12968-016-0268-8)
Supplement: Additional file 2: Table S2. — Inter-test reproducibility of peak strains from patient cohort in a previous study. (DOCX 21 kb) [file 12968_2016_268_MOESM2_ESM.docx]

**Inter-test Reproducibility of Peak Strains from Patient Cohort in a Previous Study**

In the same previous study with identical methodology, 30 patients were enrolled and were imaged twice on the same day by different technologists [1]. Inter-test reproducibility of LV and RV global and regional peak strains was assessed by Bland-Altman analyses and coefficients of variation (Table S2). For circumferential strain, each ventricle was divided into 9 regions. For longitudinal strain, each ventricle was divided into “lateral” and “septum” regions on the four-chamber image.

For circumferential strain, the Bland-Altman 95% limits of agreement were similar both globally and regionally between the two ventricles. The RV generally demonstrated higher coefficients of variation because the magnitude of circumferential strain is lower in the RV. For longitudinal strain, similar 95% limits and coefficients of variation were seen globally between the RV and LV. Regionally, the RV was more reproducible than the LV both in 95% limits and coefficient of variation. With the exception of the circumferential strain in the basal outflow region of the RV, all of the coefficients of variation were below 20%.

| **Table S2. Inter-test Reproducibility of Peak Strains** | | | |
| --- | --- | --- | --- |
|  | *Bias (%)* | *95% Limits (%)* | *CoV (%)* |
| **Circumferential Strain** |  |  |  |
| *LV - Global* | -0.9 | 4.5 | 5 |
| *RV - Global* | -0.9 | 4.4 | 8 |
|  |  |  |  |
| *LV - Basal Anterior* | -0.6 | 6.1 | 6 |
| *LV - Basal Septum* | -1.0 | 4.1 | 6 |
| *LV - Basal Inferior* | -0.6 | 5.3 | 4 |
|  |  |  |  |
| *LV - Mid Anterior* | -1.7 | 6.8 | 8 |
| *LV - Mid Septum* | -0.7 | 4.4 | 6 |
| *LV - Mid Inferior* | -0.9 | 6.1 | 6 |
|  |  |  |  |
| *LV - Apical Anterior* | -0.2 | 7.8 | 7 |
| *LV - Apical Septum* | -0.5 | 8.2 | 8 |
| *LV - Apical Inferior* | -0.5 | 8.0 | 8 |
|  |  |  |  |
| *RV - Basal Outflow* | -1.3 | 6.9 | 22 |
| *RV - Basal Septum* | -0.4 | 3.9 | 10 |
| *RV - Basal Sinus* | -1.4 | 4.4 | 7 |
|  |  |  |  |
| *RV - Mid Outflow* | -0.2 | 8.3 | 14 |
| *RV - Mid Septum* | 0.2 | 3.5 | 7 |
| *RV - Mid Sinus* | -1.1 | 6.1 | 7 |
|  |  |  |  |
| *RV - Apical Outflow* | -1.8 | 9.7 | 13 |
| *RV - Apical Septum* | -0.7 | 7.5 | 14 |
| *RV - Apical Sinus* | -2.0 | 6.4 | 7 |
|  |  |  |  |
| **Longitudinal Strain** |  |  |  |
| *LV - Global* | -0.2 | 3.7 | 7 |
| *RV - Global* | -0.1 | 4.4 | 7 |
|  |  |  |  |
| *LV - Lateral* | -2.2 | 7.6 | 17 |
| *LV - Septum* | 1.9 | 7.0 | 11 |
|  |  |  |  |
| *RV - Lateral* | 0.1 | 5.8 | 11 |
| *RV - Septum* | -0.3 | 5.2 | 8 |

**References:**

1. Jing L, Haggerty CM, Suever JD, Alhadad S, Prakash A, Cecchin F, Skrinjar O, Geva T, Powell AJ, Fornwalt BK: **Patients with repaired tetralogy of Fallot suffer from intra- and inter-ventricular cardiac dyssynchrony: a cardiac magnetic resonance study.** *Eur Heart J Cardiovasc Imaging* 2014, **15**:1333–43.
