# Supplementary material for: Left and right ventricular dyssynchrony and strains from cardiovascular magnetic resonance feature tracking do not predict deterioration of ventricular function in patients with repaired tetralogy of Fallot
Source: J Cardiovasc Magn Reson. 2016 Aug 22;18:49. doi: 10.1186/s12968-016-0268-8 (PMC4993000; doi:10.1186/s12968-016-0268-8)
Supplement: Additional file 3: Tables S3-S6. — Multivariate analysis with regional strains. (DOCX 28 kb) [file 12968_2016_268_MOESM3_ESM.docx]

**Multivariate Analyses with Regional Strains**

Peak regional strains at baseline are summarized in Table S3. The LV and RV were divided into the same regions in which inter-test reproducibility was assessed as shown in the previous section. To perform multivariate analyses with regional strains, we replaced the 4 global strains in the original analyses with the 22 regional strains. The correlations of regional strains with outcomes before adjusting for confounders are shown in Table S4. Several regional LV circumferential strains were related to changes in RVEF and LVEF, which is consistent with the same observation about global LV circumferential strain in the main manuscript. After adjusting for confounders and applying backward elimination, the only regional strain remaining in the model was circumferential strain in the basal outflow region of the RV. There was a weak positive correlation between circumferential strain in this region (RV – Basal Outflow) and ∆RVEF (β = 0.04, p = 0.01) (Table S5). Compared to the original multivariate analysis that used global strains, this new model was able to explain 1% more of the variability in each of the outcome variables (for a total of 2%, 18% and 10% of the variability in ∆RVEDVi, ∆RVEF and ∆LVEF, respectively). Comparisons of regional strains between “deterioration” and “no deterioration” groups are shown in Table S6. While four regional strains were significantly different between the two groups, circumferential strain in the basal outflow region of the RV was not among them.

| **Table S3. Baseline Regional Strains (n = 153)** | | |  |
| --- | --- | --- | --- |
|  |  | *Mean* ± *SD (%)* |  |
| **Circumferential Strain** | |  |  |
| *LV - Basal Anterior* |  | 27 ± 4 |  |
| *LV - Basal Septum* |  | 22 ± 4 |  |
| *LV - Basal Inferior* |  | 32 ± 4 |  |
|  |  |  |  |
| *LV - Mid Anterior* |  | 25 ± 4 |  |
| *LV - Mid Septum* |  | 22 ± 4 |  |
| *LV - Mid Inferior* |  | 28 ± 4 |  |
|  |  |  |  |
| *LV - Apical Anterior* |  | 30 ± 5 |  |
| *LV - Apical Septum* |  | 24 ± 5 |  |
| *LV - Apical Inferior* |  | 30 ± 5 |  |
|  |  |  |  |
| *RV - Basal Outflow* |  | 9 ± 6 |  |
| *RV - Basal Septum* |  | 11 ± 4 |  |
| *RV - Basal Sinus* |  | 18 ± 4 |  |
|  |  |  |  |
| *RV - Mid Outflow* |  | 17 ± 7 |  |
| *RV - Mid Septum* |  | 14 ± 3 |  |
| *RV - Mid Sinus* |  | 22 ± 4 |  |
|  |  |  |  |
| *RV - Apical Outflow* |  | 25 ± 6 |  |
| *RV - Apical Septum* |  | 17 ± 4 |  |
| *RV - Apical Sinus* |  | 26 ± 4 |  |
|  |  |  |  |
| **Longitudinal Strain** |  |  |  |
| *LV - Lateral* |  | 16 ± 4 |  |
| *LV - Septum* |  | 23 ± 4 |  |
|  |  |  |  |
| *RV - Lateral* |  | 20 ± 4 |  |
| *RV - Septum* |  | 26 ± 4 |  |

| **Table S4. Correlation between regional strains and outcomes before adjusting for confounders (n = 153)** | | | | | | |
| --- | --- | --- | --- | --- | --- | --- |
|  | ∆RVEDVi | | ∆RVEF | | ∆LVEF | |
|  | β | *p* | β | *p* | β | *p* |
| **Circumferential Strain** |  |  |  |  |  |  |
| *LV - Basal Anterior* | 0.02 | 0.11 | **-0.05** | **0.02** | -0.05 | 0.06 |
| *LV - Basal Septum* | 0.003 | 0.78 | **-0.04** | **0.03** | **-0.08** | **0.001** |
| *LV - Basal Inferior* | 0.02 | 0.15 | **-0.06** | **<0.001** | **-0.06** | **0.009** |
|  |  |  |  |  |  |  |
| *LV - Mid Anterior* | 0.004 | 0.74 | -0.03 | 0.11 | **-0.06** | **0.004** |
| *LV - Mid Septum* | 0.003 | 0.81 | **-0.04** | **0.03** | **-0.08** | **<0.001** |
| *LV - Mid Inferior* | 0.02 | 0.17 | **-0.07** | **<0.001** | **-0.08** | **<0.001** |
|  |  |  |  |  |  |  |
| *LV - Apical Anterior* | 0.01 | 0.37 | **-0.05** | **0.008** | **-0.07** | **0.002** |
| *LV - Apical Septum* | 0.003 | 0.81 | -0.03 | 0.09 | **-0.08** | **<0.001** |
| *LV - Apical Inferior* | 0.01 | 0.25 | **-0.06** | **0.002** | **-0.06** | **0.006** |
|  |  |  |  |  |  |  |
| *RV - Basal Outflow* | 0.007 | 0.57 | 0.001 | 0.98 | -0.002 | 0.93 |
| *RV - Basal Septum* | 0.01 | 0.34 | -0.01 | 0.56 | -0.01 | 0.56 |
| *RV - Basal Sinus* | 0.007 | 0.6 | **-0.05** | **0.02** | -0.04 | 0.09 |
|  |  |  |  |  |  |  |
| *RV - Mid Outflow* | 0.02 | 0.07 | **-0.06** | **0.004** | -0.03 | 0.16 |
| *RV - Mid Septum* | 0.01 | 0.39 | -0.03 | 0.09 | -0.03 | 0.16 |
| *RV - Mid Sinus* | 0.006 | 0.59 | **-0.05** | **0.01** | **-0.05** | **0.02** |
|  |  |  |  |  |  |  |
| *RV - Apical Outflow* | 0.02 | 0.06 | -0.03 | 0.13 | -0.02 | 0.27 |
| *RV - Apical Septum* | 0.01 | 0.37 | -0.01 | 0.59 | -0.01 | 0.51 |
| *RV - Apical Sinus* | 0.002 | 0.88 | -0.03 | 0.17 | -0.03 | 0.18 |
|  |  |  |  |  |  |  |
| **Longitudinal Strain** |  |  |  |  |  |  |
| *LV - Lateral* | 0.0001 | 0.99 | -0.02 | 0.23 | -0.03 | 0.16 |
| *LV - Septum* | 0.02 | 0.12 | **-0.05** | **0.005** | -0.05 | 0.05 |
|  |  |  |  |  |  |  |
| *RV - Lateral* | 0.002 | 0.87 | -0.04 | 0.05 | **-0.04** | **0.04** |
| *RV - Septum* | -0.006 | 0.6 | -0.04 | 0.06 | -0.03 | 0.23 |

| **Table S5. Summary of Multivariate Analysis with Regional Strains** | | | | | | | | | |  |
| --- | --- | --- | --- | --- | --- | --- | --- | --- | --- | --- |
|  | ∆RVEDVi | | | ∆RVEF | | | | ∆LVEF | | |
|  | β | *p* | | | β | *p* | β | | *p* | |
| LVEF | 0.006 | 0.62 | -0.03 | | | 0.06 | **-0.16** | | **<0.001** | |
| RVEF | 0.007 | 0.66 | **-0.17** | | | **<0.001** | -0.003 | | 0.9 | |
| RVESVi | 0.010 | 0.48 | **-0.06** | | | **0.02** | -0.04 | | 0.23 | |
| QRS Duration | -0.004 | 0.74 | -0.008 | | | 0.66 | -0.004 | | 0.88 | |
| RV – Basal Outflow | -0.002 | 0.88 | **0.04** | | | **0.01** | 0.04 | | 0.05 | |

| **Table S6. Comparison of regional strains (mean ± SD) between patients with and without deterioration.** | | | |
| --- | --- | --- | --- |
|  | Deterioration | No deterioration | p-value |
|  | (n = 37) | (n = 38) |  |
| **Circumferential Strain** |  |  |  |
| *LV - Basal Anterior* | 29 ± 4 | 27 ± 4 | **0.04** |
| *LV - Basal Septum* | 23 ± 3 | 22 ± 4 | 0.09 |
| *LV - Basal Inferior* | 33 ± 3 | 32 ± 4 | 0.07 |
|  |  |  |  |
| *LV - Mid Anterior* | 26 ± 3 | 25 ± 4 | 0.27 |
| *LV - Mid Septum* | 23 ± 4 | 22 ± 3 | 0.31 |
| *LV - Mid Inferior* | 29 ± 3 | 27 ± 4 | **0.01** |
|  |  |  |  |
| *LV - Apical Anterior* | 31 ± 4 | 29 ± 4 | **0.03** |
| *LV - Apical Septum* | 25 ± 5 | 23 ± 4 | 0.24 |
| *LV - Apical Inferior* | 32 ± 5 | 29 ± 5 | 0.05 |
|  |  |  |  |
| *RV - Basal Outflow* | 9 ± 6 | 7 ± 5 | 0.20 |
| *RV - Basal Septum* | 11 ± 4 | 10 ± 3 | 0.24 |
| *RV - Basal Sinus* | 18 ± 4 | 18 ± 4 | 0.53 |
|  |  |  |  |
| *RV - Mid Outflow* | 19 ± 7 | 16 ± 7 | 0.04 |
| *RV - Mid Septum* | 14 ± 3 | 13 ± 2 | 0.09 |
| *RV - Mid Sinus* | 23 ± 3 | 22 ± 4 | 0.14 |
|  |  |  |  |
| *RV - Apical Outflow* | 27 ± 5 | 24 ± 6 | **0.04** |
| *RV - Apical Septum* | 17 ± 3 | 16 ± 3 | 0.40 |
| *RV - Apical Sinus* | 26 ± 3 | 26 ± 3 | 0.60 |
|  |  |  |  |
| **Longitudinal Strain** |  |  |  |
| *LV - Lateral* | 16 ± 4 | 16 ± 4 | 0.69 |
| *LV - Septum* | 23 ± 4 | 22 ± 4 | 0.18 |
|  |  |  |  |
| *RV - Lateral* | 20 ± 4 | 20 ± 3 | 0.87 |
| *RV - Septum* | 26 ± 3 | 26 ± 4 | 0.82 |
